# The crystal structure of the *Yersinia pestis* iron chaperone YiuA reveals a basic triad binding motif for the chelated metal

**DOI:** 10.1107/S2059798317015236

**Published:** 2017-10-26

**Authors:** Christopher D. Radka, Dongquan Chen, Lawrence J. DeLucas, Stephen G. Aller

**Affiliations:** a Graduate Biomedical Sciences Microbiology Theme, University of Alabama at Birmingham, Birmingham, AL 35294, USA; b Comprehensive Cancer Center, University of Alabama at Birmingham, Birmingham, AL 35294, USA; c Office of the Provost, University of Alabama at Birmingham, Birmingham, AL 35294, USA; d Department of Pharmacology and Toxicology, University of Alabama at Birmingham, Birmingham, AL 35294, USA

**Keywords:** YiuA, *Yersinia pestis*, plague, transition-metal homeostasis, substrate-binding protein (SBP), X-ray crystallography, docking

## Abstract

Biological chelating molecules called siderophores are used to sequester iron and maintain its ferric state. Bacterial substrate-binding proteins (SBPs) bind iron–siderophore complexes and deliver these complexes to ATP-binding cassette (ABC) transporters for import into the cytoplasm, where the iron can be transferred from the siderophore to catalytic enzymes. In *Yersinia pestis*, the causative agent of plague, the *Yersinia* iron-uptake (Yiu) ABC transporter has been shown to improve iron acquisition under iron-chelated conditions. The Yiu transporter has been proposed to be an iron–siderophore transporter; however, the precise siderophore substrate is unknown. Therefore, the precise role of the Yiu transporter in *Y. pestis* survival remains uncharacterized. To better understand the function of the Yiu transporter, the crystal structure of YiuA (YPO1310/y2875), an SBP which functions to present the iron–siderophore substrate to the transporter for import into the cytoplasm, was determined. The 2.20 and 1.77 Å resolution X-ray crystal structures reveal a basic triad binding motif at the YiuA canonical substrate-binding site, indicative of a metal-chelate binding site. Structural alignment and computational docking studies support the function of YiuA in binding chelated metal. Additionally, YiuA contains two mobile helices, helix 5 and helix 10, that undergo 2–3 Å shifts across crystal forms and demonstrate structural breathing of the c-clamp architecture. The flexibility in both c-clamp lobes suggest that YiuA substrate transfer resembles the Venus flytrap mechanism that has been proposed for other SBPs.

## Abstract 2

YiuA, a *Yersinia pestis* substrate-binding protein, contains a putative basic triad binding motif in the canonical substrate-binding site, indicative of a metal-chelate complex binding site. Additional structural and simulation analyses support the putative function of YiuA binding bacterial siderophores.

## 1. Introduction   

Prokaryotic cells rely on ATP-binding cassette (ABC) transporters to facilitate substrate transport across cellular membranes. Bacterial ABC transporters are modular nanomachines that are crucial for cell viability and infection, and make up a superfamily of proteins composed of a cytoplasmic nucleotide-binding domain, a homodimer or heterodimer transmembrane permease and a substrate-binding protein (SBP) that localizes to the periplasm in Gram-negative bacteria or is anchored to the cell membrane in Gram-positive bacteria (Maqbool *et al.*, 2015 ▸). The genes that make up bacterial ABC transporters are genetically organized into loci that are upregulated by single promoters. These single promoters are likely to be able to simultaneously upregulate multiple genes because many of the genes contain overlapping start and stop codons, which also maintain the stoichiometry of the genes by coupling translation (Price *et al.*, 2006 ▸). In the case of iron transporters, the Fur repressor is generally considered to be the global regulator of these ABC transporter promoters (Zhou *et al.*, 2006 ▸; Han *et al.*, 2007 ▸; Gao *et al.*, 2008 ▸).

In Gram-negative bacteria, there are several genetic and structural features that are common to bacterial ABC transporters that transport metal-chelate complexes and that, when present in an ABC transporter with unknown substrate, indicate that a metal-chelate complex is indeed the transport substrate. Genetically, the genes that make up the ABC transporter are flanked by a gene encoding an outer membrane receptor for the metal-chelate complex and genes that encode biosynthetic enzymes that synthesize the sidero­phore that will complex with the metal. An example of this is the *Yersinia pestis* YsuERABCDGHIJF operon. YsuR is an outer membrane receptor, YsuABCD make up the ABC transporter and YsuEGHIJF encode siderophore-biosynthetic enzymes (Gao *et al.*, 2008 ▸). Alternatively, some ABC transporters that transport metal-chelate complexes are not adjacent to genes encoding siderophore-biosynthetic enzymes but are still adjacent to a gene encoding an outer membrane receptor. An example of this is the HmuRSTUV locus. HmuR is an outer membrane receptor, HmuS is a cytoplasmic protein that is involved in the utilization of heme-chelated iron and HmuTUV encode the ABC transporter (Thompson *et al.*, 1999 ▸). Structurally, the canonical metal-binding site of SBPs that bind metal-chelate complexes contains a basic triad binding motif made up of lysine and/or arginine amino-acid residues for interaction with the chelator. The basic triad binding motif stabilizes electrostatic interactions with the O atoms that are prevalent throughout the chelator substrate (Müller *et al.*, 2006 ▸; Peuckert *et al.*, 2009 ▸; Zawadzka *et al.*, 2009 ▸). This binding motif is chemically distinct from the binding motifs of other metal-binding SBPs, which contain glutamate, aspartate, histidine and cysteine amino-acid residues for direct interaction with metal atoms.

The *Y. pestis* *Yersinia* iron-uptake (Yiu) locus encodes an ABC transporter that is Fur-regulated and has been shown to restore the growth of an enterobactin-deficient mutant strain of *Escherichia coli* under iron-chelated conditions *in vitro* (Kirillina *et al.*, 2006 ▸). In another study, *Y. pestis* *in vitro* gene-expression data collected during growth at 26 and 37°C indicate that the *yiuA* gene is upregulated under 2,20-dipyridyl-chelated iron and nutrient-starvation conditions as well as when the Fur repressor is genetically disrupted (Han *et al.*, 2007 ▸), supporting a role for the Yiu transporter in the response to iron starvation. The Yiu locus is composed of YiuABCR, where YiuABC encode the ABC transporter with a homodimer transmembrane permease and YiuR encodes an outer membrane receptor, although curiously it is not required for Yiu-mediated iron uptake (Kirillina *et al.*, 2006 ▸). YiuABC has 35–45% sequence identity to the *Corynebacterium diphtheriae* Irp6 iron–siderophore complex uptake system, while YiuR has 45% sequence identity to the *Vibrio cholerae* enterobactin receptor IrgA and 38 and 30% sequence identity to the *E. coli* colicin I receptor and ferrienterobactin receptor FepA, respectively (Kirillina *et al.*, 2006 ▸); IrgA, the colicin I receptor and FepA are siderophore receptors.

Historically, biochemical considerations such as gene duplication and mutation have been used to explain structure–function correlations in proteins, and advances in electron microscopy, NMR spectroscopy and X-ray crystallography have strengthened this correlation by increasing the knowledgebase of protein structures in the Protein Data Bank to nearly 125 000 structures (Berman *et al.*, 2000 ▸). Bioinformatical studies of the expansion in protein three-dimensional structural determination continue to support the first principles of divergent molecular evolution, such as ancestral proteins giving rise to structurally similar descendant proteins with low sequence similarity over long evolutionary time intervals (Dokholyan & Shakhnovich, 2001 ▸), and lead to elegant solutions for organizing proteins. Classification databases such as CATH (Orengo *et al.*, 1997 ▸), DALI (Holm & Rosenström, 2010 ▸), Pfam (Finn *et al.*, 2006 ▸) and SCOP (Murzin *et al.*, 1995 ▸) have been developed to group proteins and understand their structure–function correlation by various sequence-alignment and structural alignment tech­niques. Studies grouping similar structures into a protein domain universe graph (PDUG) indicate that protein structures can be grouped according to their unique ‘functional fingerprint’, or distribution of functions within a protein structure cluster, which is evolutionarily preserved by the tight association between structure and function (Shakhnovich *et al.*, 2003 ▸).

SBPs represent a broad class of proteins with low sequence similarity and a highly conserved three-dimensional fold. SBPs are c-clamps made up of a bilobed structure interconnected by β-strand hinges, and may be grouped according a cluster system organized by unique cluster-dependent structural features (Berntsson *et al.*, 2010 ▸). In most cases, the distinct difference between clusters is the backbone connecting the two lobes. The backbone can be variable in size and made up of α-helices, loops or a combination of α-helices, loops and β-strands (Felder *et al.*, 1999 ▸). Since the cluster system is based on similarity in tertiary structure and not substrate specificity directly, there are some SBPs within the same cluster that bind different substrates (*i.e.* cluster C contains SBPs that bind amino acids, nickel and cellobiose); however, most SBPs within a cluster bind similar substrates (Berntsson *et al.*, 2010 ▸). The utilization of high structural similarity among SBPs with shared or similar substrate-binding amino-acid residues as described by Berntsson and coworkers has supported the grouping of new SBPs into their appropriate clusters with other functionally related SBPs (Marty *et al.*, 2016 ▸; Brautigam *et al.*, 2017 ▸; Parker *et al.*, 2017 ▸).

Currently there are three subclusters of SBPs that bind transition-metal atoms: cluster A-1 SBPs directly interact with transition-metal atoms, cluster A-2 SBPs interact with metal-chelate complexes and cluster D-4 SBPs only bind Fe atoms, with a subset of the SBPs utilizing synergistic anions for direct interaction with Fe atoms (Berntsson *et al.*, 2010 ▸). Cluster A SBPs contain an α-helical backbone adjoining the α/β lobes that confers rigidity to the overall structure. A key architectural difference between cluster A-1 and A-2 SBPs is the size of the substrate-binding cavity, where the smaller A-1 cavities accommodate metal ions and the larger A-2 cavities accommodate metal-chelate complexes (Scheepers *et al.*, 2016 ▸). Cluster D SBPs contain a backbone made up of short 4–5-amino-acid loops perhaps conferring flexibility (Berntsson *et al.*, 2010 ▸). Gene duplication and mutation is likely to have given rise to the SBP clusters, and in the case of cluster A-2 SBPs is likely to have enabled bacteria to utilize biosynthetic chelate intermediates for new ligand interactions, as has been described for steroid receptors (Eick & Thornton, 2011 ▸). An example of this may include *Campylobacter jejuni* CeuA, a cluster A-2 SBP which preferentially binds iron complexed with hydrolyzed enterobactin-degradation products (Raines *et al.*, 2016 ▸). Gene duplication and mutation may also have enabled multiple cluster A-2 SBPs to interact with the same ligand, as is observed for *E. coli* FebB (Chu *et al.*, 2014 ▸) and *Bacillus subtilis* FeuA (Peuckert *et al.*, 2011 ▸), both of which are capable of interaction with enterobactin despite having only 25% sequence identity.

In this report, we document the atomic three-dimensional apo structure of the Yiu SBP YiuA. We show by structural alignment that YiuA has the greatest degree of structural similarity to cluster A-2 SBPs compared with other metal-transport SBPs, and identify the amino-acid residues that are likely to form a basic triad binding motif. Additionally, we present *in silico* substrate-docking results that suggest that YiuA may be capable of binding multiple xenosiderophores.

## 2. Materials and methods   

### 2.1. Cloning and overexpression of YiuA-H_10_   

The YpCD00015516 plasmid containing the *yiuA* gene was purchased from the DNASU Plasmid Repository (Cormier *et al.*, 2010 ▸, 2011 ▸; Seiler *et al.*, 2014 ▸). The *yiuA* gene in the YpCD00015516 plasmid is identical to the *yiuA* gene in GenBank (Clark *et al.*, 2016 ▸): GenBank reference NP_670175 and UniProt reference Q8D027. The *yiuA* gene was then cloned into a standard pET-22b vector (Novagen, catalog No. 69744) using NdeI and XhoI cloning sites. In this construct, the vector containing the *yiuA* insertion also coded for a C-terminal His_10_ tag and is expressible in *E. coli*. The plasmid was recovered from ampicillin-resistant *E. coli* colonies and the DNA sequence was verified by the University of Alabama at Birmingham Heflin Center Genomics Core Laboratory. The plasmid was transformed into *E. coli* strain BL21-CodonPlus (DE3)-RIPL competent cells (Agilent Technologies, catalog No. 230280). The transformed cells were grown in lysogeny broth (LB) containing 50 µg ml^−1^ ampicillin with shaking at 225 rev min^−1^ at 37°C. When the OD_600_ reached 0.5–0.6, the temperature was decreased to 16°C and overexpression of YiuA-His_10_ was induced for 16 h with 1 m*M* isopropyl β-d-1-thiogalactopyranoside (IPTG).

### 2.2. Purification of YiuA-H_10_   

**Figure 1:**
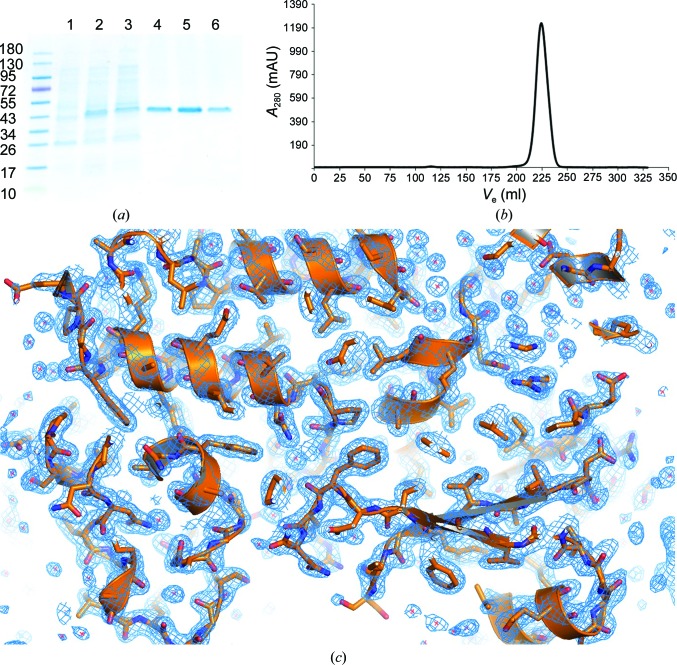
Purification and model fit of YiuA. (*a*) SDS–PAGE gel showing the enrichment of YiuA over the various steps of purification. Molecular-weight standards are shown on the left (labeled in kDa). Lane 1, uninduced BL21 whole cells. Lane 2, BL21 whole cells induced with IPTG. Lane 3, lysate supernatant fraction following French press cell disruption. Lane 4, Ni-affinity chromatography eluate. Lane 5, anion-exchange chromatography eluate. Lane 6, gel-filtration peak fraction. (*b*) HiLoad 26/600 Superdex 200 pg gel-filtration chromatogram for YiuA purification. Fractions containing the peak from this chromatogram were concentrated, are represented in lane 6 in (*a*) and were used for crystallography. (*c*) Model overlay of 2*F*_o_ − *F*_c_ electron difference density (bright blue mesh) at site 1.

YiuA-H_10_ was purified using previously described methods (Radka *et al.*, 2017 ▸), with a slight modification of the gel-filtration buffer, which consisted of 20 m*M* Tris–HCl pH 8.2, 50 m*M* NaCl, 0.05%(*w*/*v*) NaN_3_. Each purification step was monitored by SDS–PAGE (Fig. 1 ▸ *a*). The final YiuA-His_10_ purified product in gel-filtration buffer was concentrated in a centrifugal filter unit (Amicon, catalog No. UFC901024) to a final concentration of 25 ± 2 mg ml^−1^ for crystallization. In experiments designed to prolong the exposure of YiuA to metal, 1 m*M* Fe_2_(SO_4_)_3_, 1 m*M* MnCl_2_, 1 m*M* ZnCl_2_ and 1 m*M* Ga(NO_3_)_3_ were incubated with concentrated YiuA solution for 2 h prior to gel filtration, and in a separate experiment the gel-filtration column was pre-equilibrated with 1 m*M* Fe_2_(SO_4_)_3_, 1 m*M* MnCl_2_ and 1 m*M* ZnCl_2_, and YiuA with no metal supplementation was gel-filtered in gel-filtration buffer that also contained 1 m*M* Fe_2_(SO_4_)_3_, 1 m*M* MnCl_2_ and 1 m*M* ZnCl_2_.

### 2.3. Cell-viability and fractionation experiments   

*E. coli* strain BL21-CodonPlus (DE3)-RIPL competent cells (Agilent Technologies, catalog No 230280) containing the pET-22b vector (Novagen, catalog No. 69744), pYFE3 and pYIU3 plasmids were grown overnight in LB containing 50 µg ml^−1^ ampicillin with shaking at 225 rev min^−1^ at 37°C. The cells were then inoculated 1:200 into M9 minimal medium (Amresco, catalog No J863) containing 50 µg ml^−1^ ampicillin and shaking was continued at 225 rev min^−1^ at 37°C. For the Fe_2_(SO_4_)_3_ and EDDA supplementation experiments, the M9 minimal medium was equilibrated with 5 µ*M* Fe_2_(SO_4_)_3_ or 1 m*M* EDDA prior to inoculation. The fractionation protocol was as described previously (Radka *et al.*, 2017 ▸).

### 2.4. YiuA crystallization   

The YiuA-LE-H_10_ artificial residues that were added for purification remained for crystallization. Crystallization conditions were determined by optimizing initial hits that were identified by a rational approach comparing the crystallization conditions of YiuA orthologs in the Protein Data Bank (Berman *et al.*, 2000 ▸). YiuA-His_10_ crystals were grown by the hanging-drop and sitting-drop vapor-diffusion methods at 293 K in 20–25%(*w*/*v*) PEG 3350, 10 m*M* MES pH 5.5. The final, optimized condition that led to the highest resolution data set was 10 m*M* MES pH 5.5, 20%(*w*/*v*) PEG 3350. Drops consisted of YiuA-His_10_ plus reservoir solution in 1:1, 1:2 and 2:1 ratios for hanging-drop setup. Crystals were directly flash-cooled in liquid nitrogen prior to X-ray data collection. Co-crystallization experiments included separately adding 10 m*M* ZnCl_2_, 10 m*M* MnCl_2_, 1 m*M* Ga(NO_3_)_3_ or 10 m*M* Fe_2_(SO_4_)_3_ to the crystallization-drop solution. Separate crystal-soaking experiments with YiuA-H_10_ included 3 and 4 h soaks in 10 m*M* ZnCl_2_, 10 m*M* MnCl_2_, 1 m*M* Ga(NO_3_)_3_ or 10 m*M* Fe_2_(SO_4_)_3_. Some crystals changed from clear to yellow during iron-soaking experiments, although no appreciable iron anomalous signal was observed in the data.

### 2.5. X-ray data collection, structure solution and refinement   

Diffraction data were collected at 100 K on the General Medical Sciences and Cancer Institutes Structural Biology Facility (GM/CA) 23-ID-B and 23-ID-D beamlines at the Advanced Photon Source (APS), Argonne National Laboratory, Lemont, Illinois, USA. The data-collection strategy for each crystal was determined by the *iMosflm* strategy function, targeting ≥90% completeness for X-ray scattering data. The data were merged and scaled using *HKL*-2000 (Otwinowski & Minor, 1997 ▸). The data completeness and *R*_merge_ were used to determine the resolution limit. Phases were determined by MR using *Phaser* (McCoy *et al.*, 2007 ▸) as implemented in the *PHENIX* suite (Adams *et al.*, 2010 ▸). Model building and refinement were performed using *AutoBuild* in *PHENIX*. After each iteration of refinement, the structure and electron density were visualized and manually evaluated in *Coot* (Emsley *et al.*, 2010 ▸). Water molecules were incorporated automatically by *phenix.refine*. The figures were generated using *PyMOL* (v.1.8; Schrödinger; http://www.pymol.org).

### 2.6. *In silico* analyses   

#### 2.6.1. Sequence and structural alignment   

Sequence alignment of YiuA with established *Y. pestis* SBP orthologs was performed using *Clustal Omega* (http://www.ebi.ac.uk/Tools/msa/clustalo/). Sequence identities and similarities were calculated using *SIAS Sequence Identity And Similarity* (http://imed.med.ucm.es/Tools/sias.html). Structural alignment, *Q*-score, *Z*-score and r.m.s.d. were calculated by submitting the PDB codes of SBPs of interest and the atomic coordinates of molecule *B* from YiuA crystal form 1 to the *PDBeFold* server (Krissinel & Henrick, 2004 ▸; http://www.ebi.ac.uk/msd-srv/ssm/cgi-bin/ssmserver).

#### 2.6.2. Cavity measurements   

The amino-acid residues that define the YiuA cavity were predicted by uploading the atomic coordinates of molecule *B* from YiuA crystal form 1 to the *BetaCavityWeb* server (Kim *et al.*, 2015 ▸; http://voronoi.hanyang.ac.kr/betacavityweb) with a solvent radius input of 1.4 Å and selecting the Lee–Richards (solvent-accessible) cavity option. The atomic coordinates were then read in *PyMOL* and residues were manually selected based on channel-defining residues from the *BetaCavityWeb* log file. The cavity residues and site 1 residues were defined as unique objects in *PyMOL* and additional parameters were defined (dot_density, 4; dot_solvent, 1). Solvent-exposed surface areas were calculated using the *PyMOL* get_area command (*i.e.* get_area cavity).

#### 2.6.3. Substrate docking   

The substrate library of potential YiuA substrates was compiled from a combination of sidero­phores, small molecules and sideromycins from the PubChem database (Kim *et al.*, 2016 ▸), as well as siderophores and small molecules in the PDB (Berman *et al.*, 2000 ▸) that have previously been co-crystallized with cluster A-2 SBPs. *AutoDockTools* 4.2 were used for substrate-library docking simulations and analyses (Morris *et al.*, 2009 ▸). Separate simulations were run using molecules *A* and *B* from YiuA crystal forms 1 and 2 as receptors.

#### 2.6.4. Transcription-factor binding-site mapping   

Transcription-factor binding-site identification, consensus binding-site identificaion and comparison of different ABC transporter promoter sequences were conducted using the *Virtual Footprint Promoter Analysis* server (Münch *et al.*, 2005 ▸; http://www.prodoric.de/vfp/vfp_promoter.php). Promoter sequences that were submitted to the server were obtained from the GenBank (Clark *et al.*, 2016 ▸) *Y. pestis* reference genome sequence NC_003143.1.

## 3. Results   

### 3.1. In-depth molecular-replacement structure determination of YiuA   

YiuA (YiuA-H_10_) was isolated to apparent purity (>95%) and homogeneity by nickel-affinity, anion-exchange and gel-filtration chromatography (Figs. 1 ▸ *a* and 1 ▸ *b*). YiuA migrates as a single 44 kDa band on an SDS–PAGE gel (Fig. 1 ▸ *a*). The crystallization condition that led to diffraction-quality crystals was identified by optimization of initial hits from rational screening, and enabled atomic resolution X-ray crystallo­graphy (Fig. 1 ▸ *c*). Initially, crystals that grew within three weeks diffracted to approximately 2.20 Å resolution and the Bravais lattice belonged to the orthorhombic crystal system *P*2_1_2_1_2_1_. The YiuA crystals that belonged to this crystal system had unit-cell parameters *a* = 41, *b* = 95, *c* = 172 Å and will be referred to as crystal form 1. After several months of incubation, crystallization drops that had previously been clear grew crystals that diffracted to approximately 1.77 Å resolution, and the Bravais lattice of these crystals belonged to the monoclinic crystal system *P*12_1_1. The YiuA crystals that belonged to this crystal system had unit-cell parameters *a* = 46, *b* = 97, *c* = 74 Å and will be referred to as crystal form 2.

To perform molecular replacement (MR) and attempt to solve the structure of YiuA crystal form 1, we searched the YiuA amino-acid sequence against the PDB using the ‘Sequences search’ function and found that *Veillonella parvula* FepB (PDB entry 4mo9; Midwest Center for Structural Genomics, unpublished work) had the highest amino-acid sequence identity (29%) to YiuA. We used the 4mo9 coordinates in a fast MR search method with additional input parameters of a high-resolution limit of 4 Å, an r.m.s.d. variance of 3 Å, two copies of the search molecule and no alternative space group to *P*2_1_2_1_2_1_. Using these constraints resulted in an MR solution with a log-likelihood gain (LLG) score of 47.89 and a translation-function *Z* (TFZ) score of 7.8. Using this MR solution, we attempted automated model building using *AutoBuild* in *PHENIX* (Adams *et al.*, 2010 ▸) with additional input parameters of rebuild in place FALSE, six refinement cycles, 15 maximum iterative build cycles, 25 maximum iterative rebuild cycles, unchecked input model refinement before rebuilding and unchecked keep input ligands. Using these constraints resulted in 93% of the amino-acid residues being built and a post-*AutoBuild* refinement with an *R*_work_ and *R*_free_ of 24 and 30%, respectively. The remaining residues were built manually in *Coot*, and the model was refined to an *R*_work_ and *R*_free_ of 20 and 25%, respectively. The structural model of YiuA crystal form 2, produced using YiuA crystal form 1 as a starting template, was refined to an *R*_work_ and *R*_free_ of 19 and 22%, respectively. Data statistics are provided in Table 1 ▸.

**Figure 2:**
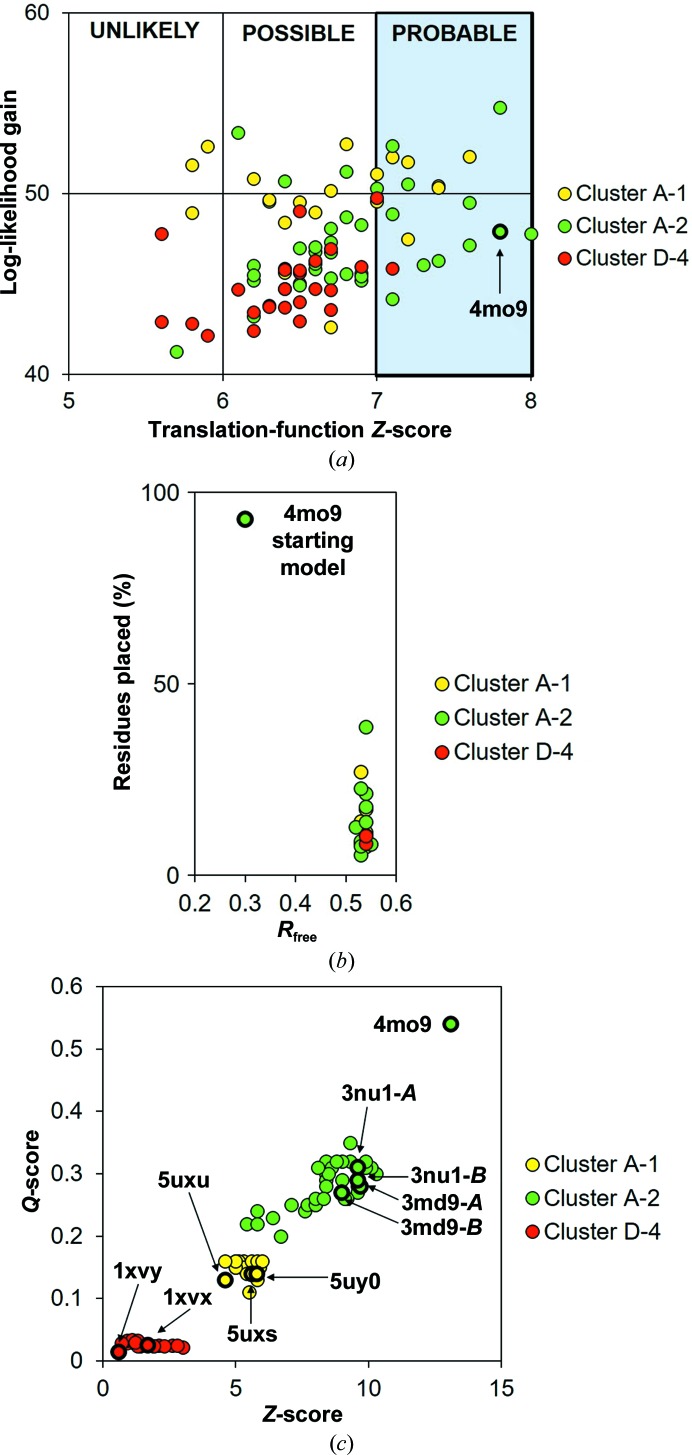
Structure determination of YiuA and SBP alignment. (*a*) Scatterplot of MR scores from a brute-force search using cluster A-1, A-2 and D-4 SBP search models. Several search models achieved MR scores that suggest a probable solution, represented by data points in the shaded panel. PDB entry 4mo9, the only search model that successfully phased the YiuA data, is notated. (*b*) Brute-force automated model building using all starting models that achieved probable MR scores. Only the starting model from the 4mo9 search built >50% of the YiuA amino acids. (*c*) Secondary-structural alignment of YiuA against all SBPs used in (*a*). Alignments are scored by *PDBeFold* *Q*-score and *Z*-score algorithms. PDB entry 4mo9 is the most structurally similar SBP to YiuA of the test set. Other *Y. pestis* SBPs are labeled as well: YfeA (PDB entries 5uxs, 5uxu and 5uy0), HmuT (PDB entries 3md9 and 3nu1) and YfuA (PDB entries 1xvx and 1xvy). The HmuT results are also denoted with *A* for molecule *A* and *B* for molecule *B*.

Next, we tested the hypothesis that other metal-binding SBPs belonging to the same cluster could be used to phase the YiuA electron-density data and solve the structure by MR. To do this, we compiled a library of cluster A-1, A-2 and D-4 SBPs modeled from the SBP structural distance tree from Berntsson *et al.* (2010 ▸) and used a brute-force MR approach, searching each SBP with the same additional input parameters as used with PDB entry 4mo9. To date, more cluster A-2 SBPs have been structurally determined than cluster A-1 or D-4, causing the sampling of cluster A-2 SBPs in this experiment to exceed those of cluster A-1 or D-4 SBPs. In cases where the search model contained multiple molecules, we searched for each molecule separately. We evaluated whether MR had potentially solved the structure using the following criteria as described on the PhaserWiki (http://www.phaser.cimr.cam.ac.uk/index.php/Molecular_Replacement). TFZ score: TFZ < 5 = no; 5 < TFZ < 6 = unlikely; 6 < TFZ < 7 = possibly; 7 < TFZ < 8 = probably; TFZ > 8 = definitely. LLG score: all tested metal-binding SBP input models returned a TFZ between 5 and 8 and an LLG score between 40 and 60 (Fig. 2 ▸ *a*), suggesting that any of the search models may have possibly phased the YiuA electron-density data. This also indicated that the LLG score would not be a useful discriminant for further analysis as all LLG scores were in the possibly solved category. Eight of 23 cluster A-1 SBP search models returned a TFZ greater than or equal to 7, including *Y. pestis* YfeA (PDB entry 5uxs; Radka *et al.*, 2017 ▸), which returned a TFZ of 7.2. 12 of 36 cluster A-2 SBP search models returned a TFZ greater than or equal to 7, including *Y. pestis* HmuT (PDB entry 3md9 molecule *B*; Mattle *et al.*, 2010 ▸). Two of 26 cluster D-4 SBP search models returned a TFZ greater than or equal to 7. The *Y. pestis* cluster D-4 SBP YfuA (PDB entry 1xvx; Shouldice *et al.*, 2005 ▸) returned a TFZ of 5.6. To determine whether any of the MR searches were successful, we took an automated model-building brute-force approach, autobuilding YiuA from each input model, which resulted in a TFZ greater than or equal to 7, with the same input parameters as used with 4mo9. In all cases except 4mo9, the *R*_free_ of the overall best autobuilt model was greater than 50%, fewer than 40% of the amino-acid residues were built and the resulting 2*F*_o_ − *F*_c_ electron-density maps were uninterpretable (Fig. 2 ▸ *b*). The only MR solution that was truly successful and led to a complete model came from 4mo9, which is a cluster A-2 SBP whose substrate is heme and enterobactin siderophore.

### 3.2. YiuA is a c-clamp and is not likely to bind free transition-metal ions   

**Figure 3:**
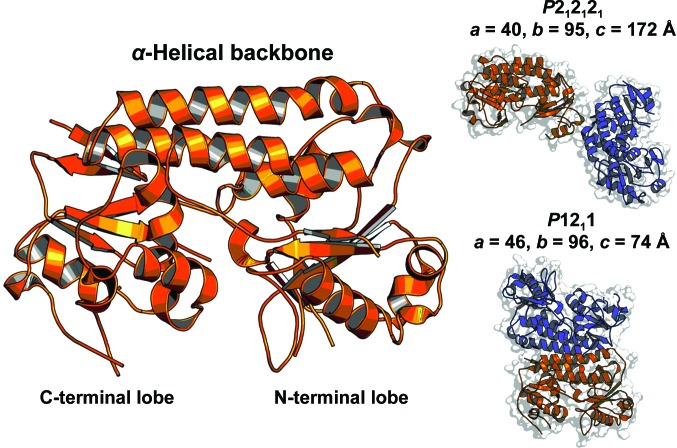
Overall structure of YiuA. Cartoon representation of the YiuA c-clamp architecture. The structure contains two globular lobe domains that are interconnected by an α-helical backbone. On the right, the orientation of the two molecules in the asymmetric unit is shown for each crystal form.

**Figure 4:**
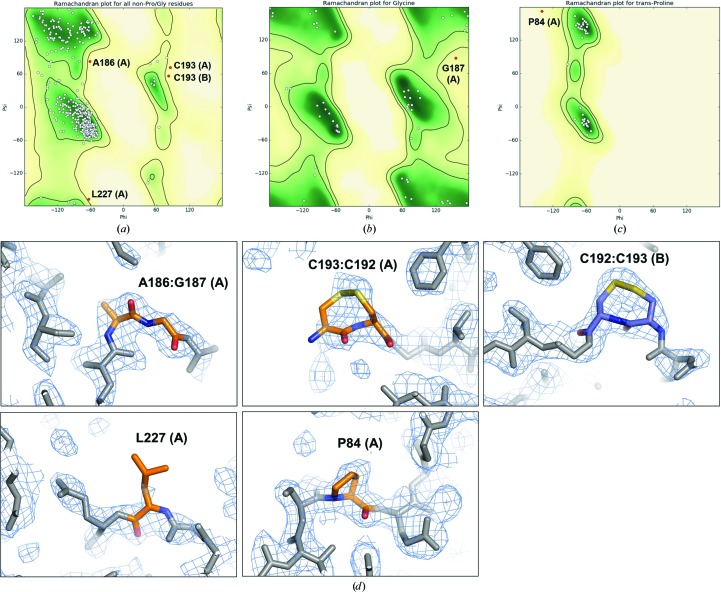
Ramachandran outliers in crystal form 1. (*a*, *b*, *c*) Ramachandran plots identifying outliers in the crystal form 1 model. (*d*) Model overlay of 2*F*_o_ − *F*_c_ electron difference density (bright blue mesh) at each outlier residue shows that the model is justified at these positions.

**Figure 5:**
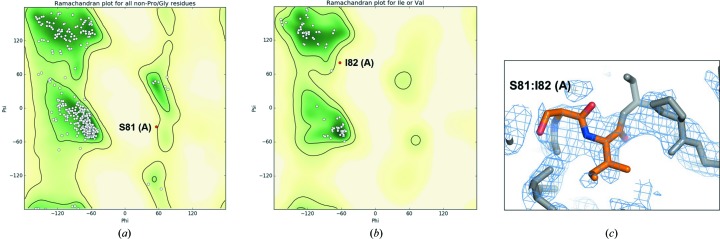
Ramachandran outliers in crystal form 2. (*a*, *b*) Ramachandran plots identifying outliers in the crystal form 2 model. (*c*) Model overlay of 2*F*_o_ − *F*_c_ electron difference density (bright blue mesh) at each outlier residue shows that the model is justified at these positions.

In both crystal forms, YiuA folds into the evolutionarily conserved c-clamp with amino-terminal and carboxy-terminal α/β globular domains joined by an α-helical backbone linking region (Fig. 3 ▸) indicative of a cluster A SBP (Scheepers *et al.*, 2016 ▸). Each α/β domain–backbone interface is defined by β-strand hinges, a feature that is also found at the α/β domain–backbone interface in other SBPs. The final refinement of both YiuA crystal form models revealed several amino-acid Ramachandran outliers. In crystal form 1 the Ramachandran outliers are Pro84, Ala186, Gly187, Cys193 and Leu227 in molecule *A*, and Cys193 in molecule *B* (Fig. 4 ▸). In crystal form 2 the Ramachandran outliers are Ser81 and Ile82 in molecule *A* (Fig. 5 ▸). The amino-acid numbering is based on the model. Close inspection of these residues shows that they have well defined corresponding electron density and are correctly modeled, even though they do not adopt idealized geometry. We also noticed that the two cysteine residues in the YiuA amino-acid sequence form an intramolecular disulfide bond at the base of the carboxy-terminal lobe in both YiuA crystal forms, perhaps to stabilize the lobe base or present a recognition motif to the YiuBC transporter for interaction. We have recently proposed an asymmetrical mechanism for YfeA substrate transfer in view of YfeA containing a rigid lobe and a flexible lobe that may undergo structural rearrangement during substrate transfer (Radka *et al.*, 2017 ▸). The YiuA disulfide bond may also play an asymmetrical role in substrate transfer.

YiuA is in the apo form in both crystal forms, therefore the canonical substrate-binding site is not as clearly distinguishable as is the case in other SBPs such as YfeA, where strong metal anomalous signal unmistakably designates the location of the YfeA canonical substrate-binding site (Radka *et al.*, 2017 ▸). Initially, we attempted to co-crystallize YiuA with manganese, zinc, iron and gallium, although those efforts failed to reveal any bound metal ions in the resulting crystal structures. We also soaked YiuA crystals in manganese, zinc, iron and gallium, still revealing no bound metals. Gallium was included in these experiments as a redox-inactive ferric iron mimic since gallium has been shown to be a suitable substrate for iron-binding proteins (Chitambar, 2016 ▸). We considered that longer exposure to metals over the course of purification may be required to load YiuA with substrate, so we attempted to co-purify YiuA with metals individually and collectively by concentrating YiuA in the presence of metal(s) prior to gel filtration, and including metal(s) in the gel-filtration buffer itself. These experiments included variance in metal counterions (*i.e.* chloride, nitrate and sulfate), as well as testing both ferrous (maintained by reducing agent) and ferric iron. Similar methods have previously been described to successfully reconstitute a holo (metal-bound) SBP from an apo SBP (Couñago *et al.*, 2014 ▸; Handali *et al.*, 2015 ▸; Vigonsky *et al.*, 2015 ▸); however, all these efforts failed to produce holo YiuA, suggesting that the YiuA substrate is not solely a metal atom.

### 3.3. YiuA has the greatest structural similarity to cluster A-2 SBPs   

The *Y. pestis* iron transporters Yfe (Bearden *et al.*, 1998 ▸; Bearden & Perry, 1999 ▸; Desrosiers *et al.*, 2010 ▸; Perry *et al.*, 2012 ▸), Hmu (Hornung *et al.*, 1996 ▸; Thompson *et al.*, 1999 ▸; Rossi *et al.*, 2001 ▸) and Yfu (Gong *et al.*, 2001 ▸; Kirillina *et al.*, 2006 ▸) have been characterized, and related SBPs have been structurally determined. YfeA (Radka *et al.*, 2017 ▸), HmuT (Mattle *et al.*, 2010 ▸) and YfuA (Shouldice *et al.*, 2005 ▸) represent SBP clusters A-1, A-2 and D-4, respectively. YiuA has approximately 19% sequence identity to YfeA, 17% sequence identity to HmuT and 16% sequence identity to YfuA. To test the hypothesis that YiuA is most structurally similar to the cluster A-2 SBPs and to broaden the structural alignment analysis to include cluster A-1, A-2 and D-4 SBPs from other species, we compiled an SBP model library based on previous SBP clustering (Berntsson *et al.*, 2010 ▸) and used *PDBeFold* (Krissinel & Henrick, 2004 ▸) to provide a more rigorous assessment of structural similarity. An advantage of using *PDBeFold* for structural alignment analysis is the provision of the *Q*-score, which describes the quality of the alignment normalized by the r.m.s.d. and the number of aligned residues, and the *Z*-score, which describes the statistical significance of the alignment (Krissinel & Henrick, 2004 ▸). Higher values of each statistic indicate stronger structural similarity and higher quality alignment between the subject and each query. The results from aligning each model with the YiuA crystal form 1 model are summarized in Fig. 2 ▸(*c*), and show that YiuA is most structurally similar to cluster A-2 SBPs and least structurally similar to cluster D-4 SBPs. Each SBP result appears to group with the results from other SBPs within the same cluster (*i.e.* the results from cluster D-4 SBPs group with the results from other cluster D-4 SBPs and do not group with the results from cluster A-1 or A-2 SBPs), highlighting the relative consistency in the results across clusters. YiuA has the highest quality structural alignment with PDB entry 4mo9, the only MR search model that successfully phased the YiuA electron density, although it is striking how great the gap is between PDB entry 4mo9 and the rest of the cluster A-2 SBPs. This gap may explain why the rest of the SBP search models tested could not solve the YiuA structure.

### 3.4. YiuA contains a basic triad binding motif and a large potential substrate cavity   

**Figure 6:**
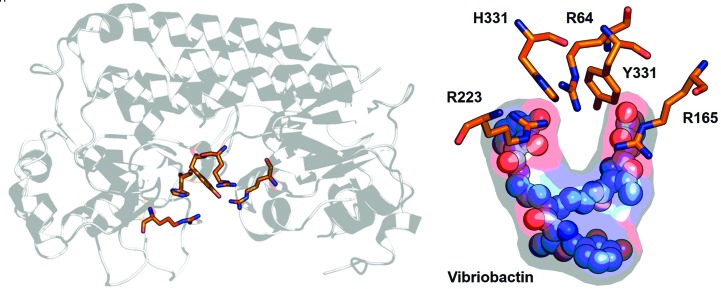
Putative YiuA site 1 amino-acid residues. The basic triad binding motif and auxiliary residues are shown, emphasizing their location at the arch of the c-clamp. A representative vibriobactin docking result demonstrates how site 1 residues might interact with a metal-chelate cargo. In this simulation, arginine and histidine residues shield substrate O atoms as the tyrosine residue is positioned to coordinate an Fe atom.

The SBP c-clamp contains a solvent-exposed cavity that spans from the arch of the c-clamp to the base of the lobes, and a substrate-binding site that resides in a pocket entombed in the cavity. The substrate-binding site, referred to as site 1, is made up of amino acids that are electronically and/or geometrically configured to bind substrate(s), and the identities of these amino acids correspond to the substrates that they bind. In the case of cluster A-1 and D-4 SBPs, nucleophilic site 1 residues such as aspartates, glutamates and cysteines are bundled together and deprotonated by histidine residues for metal binding. A survey of the YiuA cavity reveals that YiuA is void of a bundle of cluster A-1 or D-4 site 1 residues, but does contain a grouping of residues that resemble a cluster A-2 basic triad motif. These residues include Arg64, Arg165 and Arg223, and are likely to define YiuA site 1 (Fig. 6 ▸). The amino-acid numbering is based on UniProt reference sequence Q8D027. Additional interesting residues that are adjacent to the basic triad are His331 and Tyr334, which may be involved in auxiliary substrate inter­actions. A YiuA site 1 that is configured for metal-chelate complexes is not capable of high-affinity direct interaction with free metal ions, which may explain why the YiuA atomic structure does not contain evidence of ordered gallium, manganese, iron or zinc metal atoms.

Next, we compared the dimensions of the YiuA cavity with those of the YfeA and HmuT cavities. The atomic coordinates were analyzed by *BetaCavityWeb* (Kim *et al.*, 2015 ▸) to predict the residues that line the cavities of each SBP, visualized and manually refined in *PyMOL* and then measured by *PyMOL*. The total solvent-accessible surface area (SASA) of one molecule in YiuA crystal form 1 is 14 481 Å^2^ and the cavity SASA is 2682 Å^2^. The total SASA of HmuT (PDB entry 3md9; Mattle *et al.*, 2010 ▸) is 12 143 Å^2^ and the cavity SASA is 2641 Å^2^. The total SASA of YfeA (PDB entry 5uxs; Radka *et al.*, 2017 ▸) is 12 545 Å^2^ and the cavity SASA is 517 Å^2^. Based on these measurements, the dimensions of the YiuA cavity resemble the dimensions of the HmuT cavity, which accommodates a hemin complex, and are considerably larger than the YfeA cavity, which accommodates a metal ion.

### 3.5. Flexibility in the YiuA lobes widens the YiuA cavity in crystal form 2   

**Figure 7:**
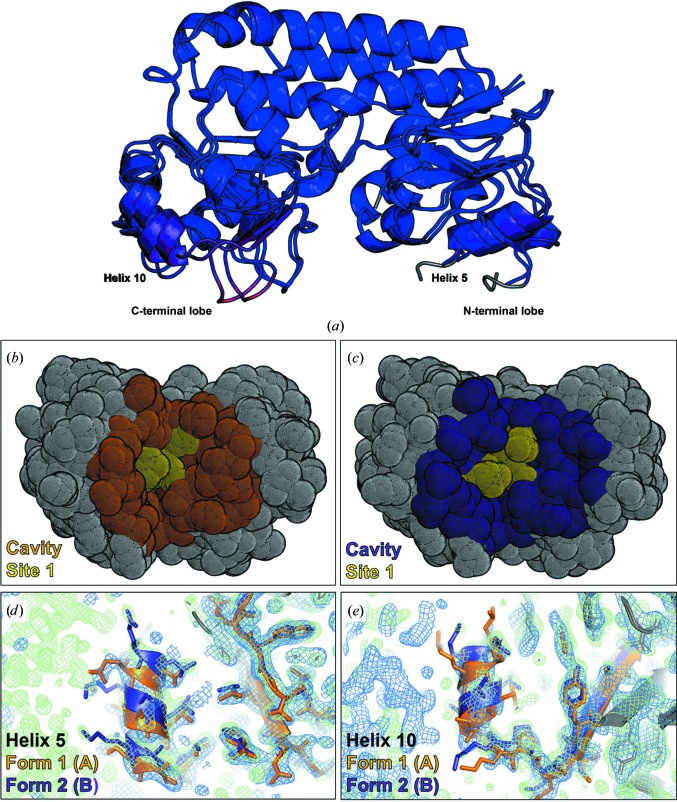
YiuA conformational changes. Structural differences between the crystal forms suggest that the carboxy-terminal lobe is a flexible lobe. (*a*) Cartoon representation of structure superimposition of YiuA crystal forms 1 and 2. Models are colored by r.m.s.d. Blue indicates good alignment, with moderate deviations in purple and higher deviations in red. White indicates residues that were not used in alignment. The highest deviations are seen in helices 5 and 10 at the bases of the lobes. (*b*) Solvent-exposed surface dots representation of the crystal form 1 base colored by outer shell (gray), cavity (orange) and site 1 pocket (yellow). Measurements of the solvent-exposed surface area are as follows: total, 14 481 Å^2^; cavity, 2682 Å^2^; site 1 pocket, 224 Å^2^. (*c*) Solvent-exposed surface dots representation of the crystal form 2 base colored by outer shell (gray), cavity (purple) and site 1 pocket (yellow). Measurements of the solvent-exposed surface area are as follows: total, 14 810 Å^2^; cavity, 2819 Å^2^; site 1 pocket, 332 Å^2^. (*d*, *e*) Structure comparison of crystal form 1 molecule *A* and crystal form 2 molecule *B* validates the conformational changes. Crystal form 1 model (orange) overlaid with 2*F*_o_ − *F*_c_ electron difference density (bright blue mesh) and crystal form 2 model (purple) overlaid with 2*F*_o_ − *F*_c_ electron difference density (light green mesh) show regions of structural identity and regions of structural differences.

The total SASA of one molecule in YiuA crystal form 2 is 14 810 Å^2^ and the cavity SASA is 2819 Å^2^. Structural alignment of the YiuA molecules used in this analysis revealed shifts in secondary-structural elements throughout the carboxy-terminal lobe and at the base of the amino-terminal lobe. The most dramatic changes occurred in helix 5 (Leu148–Ala155) and helix 10 (Leu265–Ala271), located at the bases of the lobes. The amino-acid numbering is based on UniProt reference sequence Q8D027. For simplicity, the helix numbering is based on the *PyMOL* secondary-structure assignment despite some helices containing only three residues and residues being interpreted as a helix in one crystal form and a loop in the other. Considering crystal form 1 as a point of origin, helix 5 and helix 10 both separate 2–3 Å from their points of origin to new positions and widen the cavity in crystal form 2 (Fig. 7 ▸ *a*). Glu109 is located at the end of helix 5 and Glu228 is located at the end of helix 10. Atomic distance measurements between the Glu109 and Glu228 C^∊^ atoms, delineating the base of the cavity, increase from 36.7 Å in crystal form 1 to 40.8 Å in crystal form 2. In addition to opening the c-clamp and increasing the overall YiuA SASA and cavity SASA, the shift in helices also adjusts the site 1 pocket. The site 1 pocket SASA increases from 224 Å^2^ in crystal form 1 to 332 Å^2^ in crystal form 2 (Figs. 7 ▸ *b* and 7 ▸ *c*). As an additional line of evidence for the conformational changes between crystal forms, we used *PHENIX* Structure Comparison for parallel validation and direct comparison of electron density. The superimposed unbiased 2*F*_o_ − *F*_c_ electron-density difference maps confirmed that the conformational changes are properly modeled and valid as supported by the data (Figs. 7 ▸ *d* and 7 ▸ *e*). Considering that most of the changes observed by this analysis appeared in the carboxy-terminal lobe, it is possible that YiuA may contain a flexible (carboxy) and rigid (amino) lobe, as has previously been described (Radka *et al.*, 2017 ▸).

Both YiuA crystal forms contain two molecules in the asymmetric unit, although the arrangement of the molecules is different and may be influenced by the structural differences between the crystal forms (Fig. 3 ▸). In crystal form 1, the two YiuA molecules pack orthogonally in the asymmetric unit with few intermolecular contacts near the base of the amino-terminal lobe of one molecule and the junction between the α-helical backbone and amino-terminal lobe of the other molecule. These intermolecular contacts do not involve helix 5. In crystal form 2, the two YiuA molecules pack as mirror images with extensive intermolecular contact through­out the two α-helical backbones. The two crystal forms demonstrate the dynamic, flexible nature of YiuA as a biomolecule. In the absence of cargo, the YiuA lobes may undergo some degree of oscillation to accommodate substrates of variable size, and it is possible that the crystal forms define the boundaries of this oscillation, although it is possible that the maximum degree of oscillation may exceed what has been crystallographically observed. There is certainly architectural variability in the position and spacing between secondary-structural elements from SBP to SBP, which may generally enable the c-clamp structure to ‘breathe’ and functionally resemble a Venus flytrap (Mao *et al.*, 1982 ▸). Holo YfeA can crystallize in three crystal forms with subtle changes in secondary-structural elements, showing that SBP lobes can exhibit flexibility even when substrate is bound (Radka *et al.*, 2017 ▸). The oscillation boundary that is being sampled at the time of YiuA crystal nucleation may determine which intermolecular contacts form, as the wider c-clamp may favor backbone–backbone intermolecular contacts and the narrower c-clamp may favor lobe–backbone intermolecular contacts. In this way, the intermolecular contacts would determine how the YiuA molecules pack in the crystal and thus decide which crystal form is captured by the crystallographic snapshot.

### 3.6. *In silico* docking simulation suggests that YiuA can bind siderophores and siderophore mimics   

Given that the YiuA substrate is likely to be a metal-chelate complex, we used the structures of molecules *A* and *B* from the higher resolution YiuA crystal form 2 for docking simulation experiments to estimate whether YiuA substrates could be computationally predicted. In this simulation experiment, we used a hypothetical substrate library compiled from compounds from PubChem and ligands from the Protein Data Bank. The library contained a combination of biological siderophore molecules, siderophore components and degradation products, artificial chelators, siderophore mimics, antibiotics with siderophore moieties and other small molecules that have been co-crystallized with cluster A-2 SBPs. The interaction energies from the docking simulation are summarized in Table 2 ▸. The distance between the position of a docked potential substrate relative to the estimated center of the basic triad binding motif is included along with the molecule (*A* or *B*) into which the potential substrate was docked.

Docking simulations with an overall binding free energy (Δ*G*) of Δ*G* > 0 kcal mol^−1^ are considered to be unsuccessful and are listed as ‘rejected’ for scoring and evaluation purposes. Docking simulations with an overall Δ*G* < −9 kcal mol^−1^ are considered to be reasonable and represent plausible YiuA substrates. This interval is based on the following equation relating free energy and binding constant (Du *et al.*, 2016 ▸),
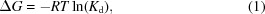
where *R* is the universal gas constant, approximated to 1.987 cal mol^−1^ K^−1^ (Wagman *et al.*, 1945 ▸), and *T* is the temperature in kelvin. In these studies, the temperature was defined as 298 K.
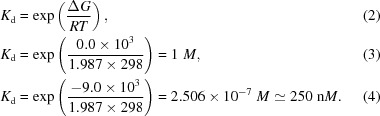
Plausible YiuA substrates include azotobactin (a siderophore from *Azotobacter vinelandii*), deferoxamine (a siderophore from *Streptomyces pilosus*), delftibactin A and B (siderophores from *Delftia acidovorans*), erythromycin (a sideromycin), ferrimycin A1 (a sideromycin), protochelin (a siderophore from *A. vinelandii*), pseudobactin (a siderophore from *Pseudomonas fluorescens*), pyoverdin and pyoverdin G4R (siderophores from *P. fluorescens*), salmycin A (sideromycin) and vibriobactin (a siderophore from *V. cholerae*). Interestingly, our results suggest that yersiniabactin (a siderophore from *Y. pestis*) is not a plausible YiuA substrate.

Representative simulation with vibriobactin demonstrates how the basic triad binding motif might enable YiuA to interact with a siderophore (Fig. 6 ▸). We hypothesize that the residues Arg64, Arg165, Arg223 and perhaps His331 act as an electrostatic touch fastener with strong positive charges that can mate with electronegative O atoms throughout the siderophore. These electrostatic interactions would maintain attachment of the substrate until its transfer to the YiuBC transporter, and may also promote direct interaction between Tyr334 and an Fe atom by reducing electronic repulsion between substrate O atoms and the YiuA tyrosyl hydroxyl. The simulations do not appear to restrict this hypothetical interaction to any specific substrate, as this interaction was predicted across all of the plausible substrates mentioned above. Future directions include screening YiuA against these plausible substrates to identify any potential physiological substrates.

### 3.7. The *Y. pestis* YiuA promoter contains numerous predicted transcription-factor binding sites that may complicate gene expression and environmental acclimation in *E. coli*   

**Figure 8:**
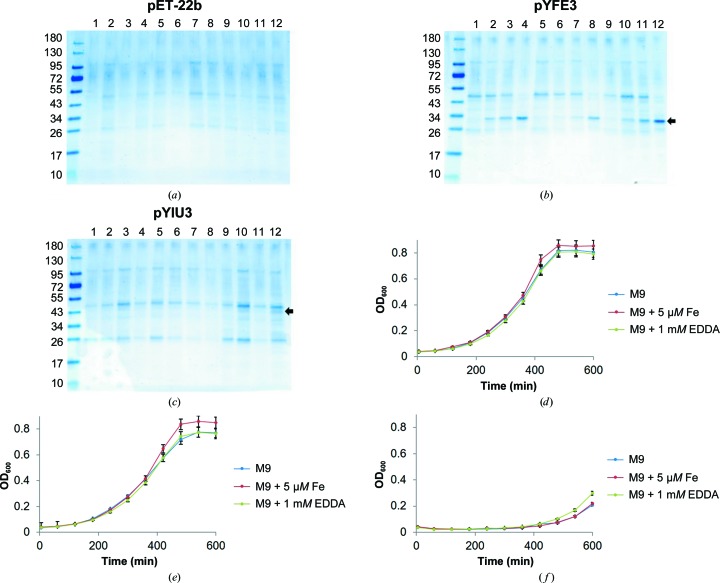
Loci acclimation *in vitro*. *E. coli* expressing the Yiu transporter require a threefold greater time to acclimate under nutrient-limiting conditions than *E. coli* expressing the Yfe transporter. (*a*, *b*, *c*) Periplasmic fractions of *E. coli* constructs growing in various *in vitro* media conditions. Black arrows denote the positions of electrophoretic migration for YfeA (*b*) and YiuA (*c*). Lanes 1–4 represent time points 3, 5, 7 and 10 h from M9 minimal medium base conditions. Lanes 5–8 represent time points 3, 5, 7 and 10 h from M9 minimal medium with 5 µ*M* Fe_2_(SO_4_)_3_ supplementation. Lanes 9–12 represent time points 3, 5, 7 and 10 h from M9 minimal medium with 1 m*M* EDDA supplementation. Molecular-weight markers are labeled in kDa. (*d*, *e*, *f*) Growth curves for the pET-22b construct (*d*), pYFE3 construct (*e*) and pYIU3 construct (*f*). Error bars represent the standard deviation in OD_600_ from experiments performed in triplicate.

Previous studies have shown that YiuA is upregulated when *Y. pestis* is grown in chemically defined minimal medium (Han *et al.*, 2007 ▸) and that recombinant Yiu transporter is functional in *E. coli* (Kirillina *et al.*, 2006 ▸); however, these studies were performed after *Y. pestis* or *E. coli* had acclimated to their environments. The *E. coli* experiments included the pYIU3 plasmid, which uses the native *Y. pestis* Yiu promoter to drive *yiu* gene expression in *E. coli* (Kirillina *et al.*, 2006 ▸). During acclimation to starvation conditions (Zhang *et al.*, 2008 ▸) or environmental stresses (Marin *et al.*, 2004 ▸), many key genetic events occur on the minute to hour timescale that uncover how organisms adjust to their environments. To examine *yiuA* gene expression in the context of environmental and genetic acclimation, we first measured cell growth and probed periplasmic fractions of an *E. coli* strain harboring the pYIU3 plasmid for YiuA. Cells were grown in chemically defined minimal medium, iron-supplemental minimal medium and minimal medium containing a metal chelator (Fig. 8 ▸). These results were compared with data obtained from an *E. coli* strain harboring an empty pET-22b vector to simulate a negative control, and an *E. coli* strain harboring the pYFE3 plasmid, which uses the native *Y. pestis* Yfe promoter to drive the expression of a recombinant Yfe transporter that has also been shown to be functional in *E. coli* (Bearden *et al.*, 1998 ▸; Bearden & Perry, 1999 ▸). *E. coli* cells containing the pYFE3 plasmid produce a substantial amount of YfeA protein that is sufficient for biophysical characterization, which is clearly apparent by SDS–PAGE (Radka *et al.*, 2017 ▸), and may serve as a positive control for recombinant *Y. pestis* SBP production driven by native promoters.

Negative control experiments indicated that *E. coli* cells containing the pET-22b vector were able to acclimate to each condition and enter exponential growth after a 2–3 h lag phase (Fig. 8 ▸ *d*). SDS–PAGE analysis of negative control periplasmic fractions showed negligible background changes in periplasmic content (Fig. 8 ▸ *a*). Positive control experiments indicated that *E. coli* cells containing the pYFE3 plasmid could acclimate to all conditions and enter exponential growth after a 2–3 h lag phase (Fig. 8 ▸ *e*). SDS–PAGE analysis of positive control periplasmic fractions showed the appearance of an ∼30 kDa band as early as 2 h after cells had acclimated to their environment and entered exponential phase growth (Fig. 8 ▸ *b*). Previous work confirmed that this band contains YfeA by mass-spectrometric analysis (Radka *et al.*, 2017 ▸). As expected, the appearance of the band containing YfeA is delayed when the cells are growing under iron-supplementation conditions and is most pronounced when the cells are growing under iron-chelated conditions, indicating the *E. coli* is responding to the Fur element in the *Y. pestis* promoter. These data show that *E. coli* cells begin producing recombinant YfeA closely following acclimation to their environment, and within 2 h of entering stationary-phase growth the band containing YfeA becomes the dominant species of the periplasmic fraction.

Outcomes with *E. coli* cells containing the pYIU3 plasmid differed from the positive and negative control results. Growth-curve experiments indicated that acclimation across all conditions occurred over a much larger timescale, as cells appeared to be arrested in a 7–8 h lag phase before eventually entering exponential phase growth (Fig. 8 ▸ *f*). This lag phase was particularly surprising considering that *E. coli* cells have been shown to exhibit doubling times of ∼1.5 h and most dramatic lag phases that lasted up to 5 h when growing in the same minimal medium as used in this study (Paliy & Gunasekera, 2007 ▸). Cells growing under iron-chelated conditions exhibited a marginal improvement in growth; however, SDS–PAGE analysis of periplasmic fractions across conditions did not show strong YiuA production (Fig. 8 ▸ *c*) such as that observed in the positive control or as would be expected based on previous reports of *yiuA* gene expression (Han *et al.*, 2007 ▸). The only periplasmic fraction to show any appreciable YiuA production was the final time point from cells growing under iron-chelated conditions (confirmed by mass-spectrometric analysis).

Next, we sought to better understand why cells containing the pYIU3 plasmid exhibited an extended lag phase. We used an informatics approach to help explain this observation in a genetic context, presuming that the growth impasse occurred from elements in the *Y. pestis* Yiu promoter given the otherwise equivalent plasmid backbones in pYFE3 and pYIU3. The *Virtual Footprint* and PRODORIC transcription-factor binding sites (TFBS) prediction tools detected several TFBS and mapped them throughout the *Y. pestis* Yiu promoter, although we limited our analysis to TFBS that would be recognized by *E. coli* and that had a hit score of at least ten to only consider the most confident predictions. 12 TFBS were predicted from both strands, with top hits including stress-related OxyR, CpxR and LexA transcription factors. By lowering the hit-score threshold, we could detect a Fur site, although its hit score was unexpectedly low considering that the Fur site in the Yiu promoter has been well characterized. We then expanded the analysis to include *Y. pestis* Hmu, Yfu, Yfe and Ysu promoters. These results are summarized in Table 3 ▸. Three, four, five and one TFBS were detected in the Hmu, Yfu, Yfe and Ysu promoters, respectively, and the only promoter that was found to contain a Fur site with a confident hit score was the Yfe promoter. Notably, the Yfe Fur site contained the highest hit score of the TFBS detected in the Yfe promoter as well as all of the TFBS detected across all of the promoters analyzed. We interpret these findings to suggest that the extensive lag time observed from *E. coli* cells harboring the pYIU3 plasmid is caused by metabolic stress from interpreting the numerous hypothetical signals encoded in the Yiu promoter. These signals may be present to regulate expression of the Yiu transporter under a specific set of conditions beyond iron starvation. Furthermore, because this information is contained in a plasmid, any potential consequences of interpreting the numerous TFBS in the genome would be expected to be exacerbated by the additional copies of the plasmid. Similar observations have been described in yeast, as genes with multiple TFBS can exhibit more variable expression patterns and contribute to slower growth (Bilu & Barkai, 2005 ▸).

## 4. Discussion   

### 4.1. Genetic experiments suggest that the YiuA substrate is chelated metal   

Iron is required for the function of the catalytic cores of many enzymes owing to the redox biochemistry that iron can perform. Iron is theorized to have been incorporated into early enzymes in the primordial earth, and as a result many of life’s metabolic pathways are configured around the properties of iron (Imlay, 2014 ▸). Owing to the central importance of iron and its requirement for survival, bacteria possess multiple, overlapping and redundant iron transporters that are generally tightly regulated by intracellular iron concentration, the ferric uptake regulator Fur and the fumarate-nitrate reduction regulator FNR (Kammler *et al.*, 1993 ▸; Troxell & Hassan, 2013 ▸; Carpenter & Payne, 2014 ▸). The study of redundant iron transporters often requires the genetic disruption of multiple transport mechanisms to detect a phenotype, evaluate its relevance in infection, assess its significance in viability and/or measure its contribution to iron transport by a specific mechanism (Perry *et al.*, 2007 ▸; Wyckoff *et al.*, 2007 ▸; Peng *et al.*, 2015 ▸). To observe iron uptake by the Yiu system, the *Y. pestis* Ybt, Yfe and Yfu transport pathways needed to be disrupted, and although the Yiu system was shown to contribute to iron uptake, YiuABC was determined to be the least effective iron-uptake transporter of the iron-uptake pathways that have been characterized (Kirillina *et al.*, 2006 ▸). Infection studies using a mouse model of bubonic plague and mutant *Y. pestis* strains with multiple disrupted iron-transport systems have determined that neither the Yfu (Gong *et al.*, 2001 ▸) nor the Yiu redundant iron transporters play a significant role in infection (Kirillina *et al.*, 2006 ▸). In another study, *Y. pestis in vivo* gene-expression data collected from plague-infected mice indicate that the *yfuA* and *yiuA* genes are downregulated during growth in the murine lung, whereas the virulence factor *yfeA* gene is upregulated (Yan *et al.*, 2013 ▸), concurring that the Yfu and Yiu transporters do not significantly contribute to the disease process.

At low pH under anaerobic conditions, ferrous iron (Fe^2+^) is soluble; however, at physiological pH under aerobic conditions ferrous iron oxidizes to insoluble particulate ferric iron (Fe^3+^) and requires tight coordination to keep the iron soluble (Wyckoff *et al.*, 2006 ▸). Bacteria utilize low-molecular-weight siderophores to chelate iron and maintain solubility, and have been shown to possess redundant siderophore-mediated iron-uptake pathways (Johnstone & Nolan, 2015 ▸). Interestingly, bacteria have also been shown to express ABC transporters to import and consume xenosiderophores, or non-native siderophores produced by competitors (Johnstone & Nolan, 2015 ▸; Peng *et al.*, 2015 ▸). Identifying which ABC transporters might be involved in the transport of a specific substrate is potentially complicated because their contribution to substrate transport may be minor relative to the contributions of other transporters, or their usefulness may require certain conditions to be met to observe activity. Databases such as TransportDB have been helpful in assigning substrates to many ABC transporters based on bioinformatics (Elbourne *et al.*, 2017 ▸); however, structural and functional data are unavailable for many of the transporters that would strengthen the reliability of the predicted substrate assignments. In TransportDB, the Yiu transporter is assigned the substrate cobalamin/Fe^3+^-siderophores because the Yiu transporter has been shown to function in iron uptake but has only been proposed to transport chelated metal (Kirillina *et al.*, 2006 ▸), as the precise substrate chelator has not yet been determined.

Gene-expression analyses of *Y. pestis* growing under a variety of selective pressures have revealed that *Y. pestis* prioritizes different iron transporters depending on environmental conditions. When *Y. pestis* is growing in human plasma, *yfeA* has the highest gene expression of the iron-transport SBPs that were detected (Rosso *et al.*, 2008 ▸), whereas when *Y. pestis* is growing under iron-starvation conditions in chemically defined medium *yiuA* has the highest gene expression of the iron-transport SBPs that were detected (Han *et al.*, 2007 ▸). This difference suggests that *Y. pestis* may prioritize the acquisition of chelated metal when growing outside a host, and the Yiu transporter significantly improves this acquisition. A selective pressure that can help to identify and target genes involved in siderophore transport are sideromycins, or antibiotics containing siderophore moieties, that parasitize siderophore-uptake systems (Braun *et al.*, 2009 ▸). Polymyxin B is a sideromycin (Suzuki *et al.*, 1993 ▸) and a potent antibiotic (Evans *et al.*, 1999 ▸; Kassamali *et al.*, 2015 ▸) that, when exposed to *Y. pestis*, drove gene expression of *hmuT*, *ysuA* and *yiuA* but triggered the downregulation of *yfeA* and *yfuA* (Han *et al.*, 2007 ▸), further supporting a role for the Yiu transporter in transporting chelated metal.

### 4.2. Atomic structures of YiuA indicate that the substrate is a chelated metal   

Ligand-binding proteins make up one of the largest functional categories in protein classification and contain many structurally similar proteins that are highly dissimilar in primary amino-acid sequence (Shakhnovich *et al.*, 2003 ▸). Structural comparisons between proteins with a ligand-binding functional fingerprint but unknown substrate against proteins with the same functional fingerprint and known substrates can confidently predict substrate identity (Maqbool *et al.*, 2015 ▸). The atomic structure of YiuA is a c-clamp with an α-helical backbone indicative of a metal-binding cluster A SBP. Structural alignment analysis reveals that YiuA has the greatest degree of structural similarity to cluster A-2 SBPs, which bind chelated metal. The discovery of a basic triad binding motif and of a cavity that is large enough to accommodate a metal-chelate complex provide additional lines of evidence, enabled by the atomic structure, that YiuA is a cluster A-2 SBP. An open question in the structural biology of metal-binding SBPs is the precise mechanism of substrate transfer. Generally, SBP substrate transfer is proposed to resemble a Venus flytrap where the c-clamp cavity opens and shuts as the lobes collapse on the substrate (Mao *et al.*, 1982 ▸). Structural comparisons of apo and holo SBPs indicate that this model is in good agreement with most SBPs (Berntsson *et al.*, 2010 ▸), with an extreme example being the LivJ cavity, which can open to 60° (Trakhanov *et al.*, 2005 ▸). In contrast, metal-binding SBPs have revealed minimal changes upon substrate binding (Lee *et al.*, 2002 ▸; Andrews *et al.*, 2003 ▸; Karpowich *et al.*, 2003 ▸; Couñago *et al.*, 2012 ▸) suggestive of a much more condensed Venus flytrap or an alternative mechanism for substrate transfer. Structural comparison of the YiuA crystal forms reveals that mobile elements in both lobes can vary the solvent-exposed surface area of the apo YiuA cavity by over 100 Å^2^. A holo YiuA structure is desired in order to shed light on remaining questions such as how much does the cavity solvent-exposed surface area change upon substrate binding, do changes to the cavity manifest in a crystal form with a new arrangement of molecules, and does the transition from apo to holo YiuA resemble a Venus flytrap?

### 4.3. The YiuA substrate could be foreign siderophores   

Protein-expression experiments indicate that *E. coli* does not respond to the Yiu promoter in the same manner as *Y. pestis*, considering that *yiuA* gene expression has been reported to exceed *yfeA* gene expression in *Y. pestis* cells growing under nutrient-starvation conditions (Han *et al.*, 2007 ▸). Instead, *E. coli* rapidly produces YfeA after acclimating to nutrient-limiting conditions in minimal medium while scarcely producing YiuA, and requires a twofold to threefold longer acclimation period before entering exponential phase growth when responding to the Yiu promoter relative to responding to the Yfe promoter. This discrepancy may be caused by a higher quantity of predicted TFBS in the Yiu promoter than are predicted in the Yfe promoter. We speculate that the numerous hypothetical TFBS in the Yiu promoter enable *Y. pestis* to utilize the Yiu transporter to respond to a limited set of specific challenges unrelated to infection, and this signaling stifles *E. coli*, preventing a quick response to *Y. pestis*-specific coding. The atomic structure of YiuA and the identification of precise amino-acid residues constituting the YiuA basic triad binding motif enabled substrate-docking simulations with a library of physiological and artificial metal chelators (Table 2 ▸). Several siderophores and sideromycins were identified as plausible YiuA substrates. Considering the absence of siderophore-biosynthetic enzymes in the Yiu locus, that any physiological Yiu substrate(s) are currently unknown and that multiple plausible substrates were predicted by substrate-docking simulations, the function of YiuR and the Yiu transporter may be to enable *Y. pestis* to utilize a wide range of siderophores including xenosidero­phores. In addition to nutrient starvation, other conditions that may promote the expression of the Yiu transporter might include growth in polymicrobial communities or coinfections. In the case of coinfections, Yiu expression could be triggered by autoinducers from other bacteria rather than host factors. Although this proposed function would suggest that YiuA is promiscuous/nonspecific for iron-chelate complexes, yersiniabactin, a *Y. pestis* siderophore, is not predicted to be a plausible YiuA substrate, suggesting the yersiniabactin periplasmic chaperone is a different SBP to YiuA. Indeed, the structures of YiuA described in this work are in the apo state despite *E. coli* producing siderophores during recombinant protein expression. Interestingly, over the course of protein purification, the YiuA-H_10_ sample assumed a rusty red shade as the sample increased in purity and concentration. This color was lost, however, when the protein sample was introduced to the crystallization condition. Efforts are ongoing to screen the plausible substrates, to achieve a holo YiuA structure, to validate the proposed function of the basic triad motif and auxiliary site 1 residues, to measure changes to the cavity and to recapitulate this tantalizing observation with a defined substrate.

## Supplementary Material

PDB reference: apo YiuA crystal form 1, 6b2x

PDB reference: crystal form 2, 6b2y

## Figures and Tables

**Table 1:** Data-collection and refinement statistics Values in parentheses are for the highest resolution shell.

Crystal	Apo YiuA crystal form 1	Apo YiuA crystal form 2
PDB code	6b2x	6b2y
Data collection		
Beamline	GM-CA, APS	GM-CA, APS
Wavelength (Å)	1.2828	1.2398
Space group	*P*2_1_2_1_2_1_	*P*12_1_1
*a*, *b*, *c* (Å)	40.57, 94.97, 171.86	46.34, 96.66, 74.19
α, β, γ (°)	90, 90, 90	90, 100.67, 90
*V*_M_ † (Å^3^ Da^−1^)	1.88	1.87
Solvent content (%)	34.72	34.18
Resolution (Å)	50.00–2.20 (2.24–2.20)	50.00–1.77 (1.80–1.77)
Unique reflections	34143 (1605)	57167 (2821)
Completeness (%)	98.6 (93.9)	90.9 (91.1)
Multiplicity	3.2 (3.0)	5.1 (5.6)
CC_1/2_	99.4 (98.4)	97.2 (97.6)
*R*_merge_ (%)	5.7 (24.8)	6.9 (32.6)
*R*_meas_ (%)	6.8 (29.8)	7.7 (36.1)
*R*_p.i.m._ (%)	3.6 (16.2)	3.3 (15.2)
Mean *I*/σ(*I*)	15.3 (3.0)	36.2 (5.0)
Refinement		
Resolution (Å)	41.47–2.20 (2.25–2.20)	45.58–1.77 (1.81–1.77)
No. of non-anomalous reflections	34079	57135
Completeness (%)	98.6 (94.6)	90.9 (91.1)
*R*_work_ (%)	19.61 (23.56)	18.60 (22.93)
*R*_free_ ‡ (%)	24.57 (31.04)	21.87 (26.14)
Wilson *B* factor (Å^2^)	35.155	27.173
Average *B* factor, overall (Å^2^)	38.42	31.16
No. of protein atoms	5151	5172
No. of solvent atoms	404 H_2_O, 2 Na, 2 Cl	616 H_2_O, 3 Na
No. of molecules in asymmetric unit	2	2
R.m.s.d.‡, bonds (Å)	0.008	0.007
R.m.s.d., angles (°)	0.992	0.856
Ramachandran plot		
Favored (%)	95.09	97.11
Allowed (%)	3.99	2.59
Outliers (%)	0.92	0.3
Clashscore	8.62	5.89
*MolProbity* score	1.83	1.48

† Matthews coefficient.

‡ The test set uses ∼5% of the data.

**Table:** Stable docking.

Substrate	PubChem/PDB	ID	Type	Energy (Δ*G*)	Distance (Å)	Molecule
Natamycin	PubChem	5281099	Sideromycin	−8.7	1.2	*A*
Yersiniabactin	PubChem	5462519	Siderophore	−8.1	2.9	*A*
Vibrioferrin	PubChem	90659786	Siderophore	−7.5	2.1	*A*
Enterobactin	PDB	EB4	Siderophore	−7.4	0.9	*A*
Enterobactin component	PDB	EHS	Siderophore component	−7.2	1.9	*A*
Enterobactin	PDB	EB4	Siderophore	−7.1	0.7	*B*
Streptomycin	PubChem	19649	Sideromycin	−6.9	1.8	*A*
Streptomycin	PubChem	19649	Sideromycin	−6.9	1.4	*B*
Vibriobactin	PubChem	56626080	Siderophore	−6.9	2.4	*A*
Vibrioferrin	PubChem	90659786	Siderophore	−6.9	1.8	*B*
Yersiniabactin	PubChem	5462519	Siderophore	−6.8	2.7	*B*
Vibrioferrin_apo	PubChem	197680	Siderophore	−6.6	0.9	*A*
MECAM	PDB	ECA	Synthetic siderophore	−6.6	2.6	*A*
4-LICAM	PDB	LCM	Synthetic siderophore	−6.4	2.2	*A*
Coprogen derivative	PubChem	102315087	Siderophore	−6.3	2.7	*B*
Enterobactin component	PDB	EHS	Siderophore component	−6.2	1.0	*B*
6-LICAM	PDB	PXJ	Synthetic siderophore	−6.2	1.5	*A*
Gentamicin	PubChem	3467	Sideromycin	−6.1	1.4	*A*
5-LICAM	PDB	5LC	Synthetic siderophore	−6.1	2.3	*A*
Schizokinen	PDB	SKZ	Siderophore	−6.1	2.5	*A*
Erythromycin	PubChem	12560	Sideromycin	−6.0	0.7	*B*
RPR209685	PDB	RRR	Small molecule	−5.9	3.6	*B*
Natamycin	PubChem	5281099	Sideromycin	−5.8	1.2	*B*
Enterobactin	PubChem	34231	Siderophore	−5.7	1.3	*A*
Deferrioxamine E	PubChem	161532	Siderophore	−5.7	3.3	*A*
Micacocidin A	PubChem	492645	Siderophore	−5.7	1.7	*A*
Pyochelin	PubChem	9973542	Siderophore	−5.7	3.6	*A*
Pyoverdin C	PubChem	102122857	Siderophore	−5.6	2.6	*B*
Vibriobactin	PDB	VBN	Siderophore	−5.5	1.8	*A*
Vibriobactin	PDB	VBN	Siderophore	−5.4	2.0	*B*
Microcin SF-608	PubChem	10793809	Sideromycin	−5.3	2.9	*A*
Ferrichrome	PDB	FCE	Siderophore	−5.2	2.6	*A*
4-LICAM	PDB	LCM	Synthetic siderophore	−5.2	2.4	*B*
Pyochelin	PubChem	9973542	Siderophore	−5.1	1.9	*B*
Pyochelin isomer	PubChem	23637949	Siderophore	−5.0	1.9	*B*
5-LICAM	PDB	5LC	Synthetic siderophore	−5.0	1.1	*B*
8-LICAM	PDB	8LC	Synthetic siderophore	−5.0	3.1	*A*
δ-2-Albomycin A1	PDB	ALB	Sideromycin	−5.0	1.0	*B*
MECAM	PDB	ECA	Synthetic siderophore	−4.9	1.6	*B*
Ferrichrome	PDB	FCE	Siderophore	−4.8	1.9	*B*
Vibrioferrin_apo	PubChem	197680	Siderophore	−4.7	2.6	*B*
Schizokinen	PDB	SKZ	Siderophore	−4.7	1.3	*B*
6-LICAM	PDB	PXJ	Synthetic siderophore	−4.6	2.3	*B*
8-LICAM	PDB	8LC	Synthetic siderophore	−4.4	2.0	*B*
Enterobactin	PubChem	34231	Siderophore	−4.3	1.1	*B*
Citric acid	PubChem	311	Small molecule	−4.2	3.5	*A*
Gentamicin	PubChem	3467	Sideromycin	−4.2	1.4	*B*
Azotochelin	PubChem	193592	Siderophore	−4.2	3.5	*A*
Alcaligin	PubChem	15090148	Siderophore	−4.2	1.7	*A*
*N*-Desferriferrichrome	PubChem	169636	Siderophore	−4.1	2.2	*B*
*N*-Desferriferrichrome	PubChem	169636	Siderophore	−4.0	1.6	*A*
Pyochelin isomer	PubChem	46173425	Siderophore	−4.0	2.9	*B*
*N* ^5^-Acetyl-*N* ^5^-hydroxy-L-ornithine	PDB	AHO	Siderophore precursor	−3.9	2.8	*B*
Alcaligin	PubChem	15090148	Siderophore	−3.8	2.0	*B*
*N* ^5^-Acetyl-*N* ^5^-hydroxy-L-ornithine	PDB	AHO	Siderophore precursor	−3.7	1.7	*A*
Rhizoferrin	PubChem	9845871	Siderophore	−3.6	1.4	*A*
Enterobactin component	PDB	DBS	Siderophore component	−3.6	2.0	*A*
Pyochelin isomer	PubChem	54579907	Siderophore	−3.5	2.0	*B*
Anguibactin	PubChem	121231152	Siderophore	−3.4	2.7	*A*
Enterobactin component	PDB	DBS	Siderophore component	−3.4	1.9	*B*
Azotochelin	PubChem	193592	Siderophore	−3.1	2.4	*B*
Micacocidin	PubChem	101948282	Siderophore	−3.1	2.1	*B*
EGTA	PubChem	6207	Small molecule	−3.0	1.5	*A*
Aerobactin	PubChem	123762	Siderophore	−2.9	2.0	*A*
Enterochelin component	PubChem	151483	Siderophore component	−2.8	1.6	*A*
Salmochelin S4	PubChem	101763507	Siderophore	−2.8	1.2	*A*
Aerobactin	PubChem	123762	Siderophore	−2.6	2.5	*B*
Staphyloferrin A	PubChem	3035516	Siderophore	−2.6	2.3	*B*
Pseudobactin	PubChem	5486206	Siderophore	−2.6	2.7	*B*
Mycobactin M	PubChem	5748534	Siderophore	−2.6	1.9	*B*
Microcin SF-608	PubChem	10793809	Sideromycin	−2.6	2.4	*B*
Staphyloferrin B	PDB	SE8	Siderophore	−2.6	1.8	*B*
Mycobactin M	PubChem	5748534	Siderophore	−2.5	2.0	*A*
Rhizoferrin	PubChem	9845871	Siderophore	−2.5	1.9	*B*
Staphyloferrin B	PDB	SE8	Siderophore	−2.5	1.8	*A*
EDDA	PubChem	61975	Small molecule	−2.4	2.2	*A*
Rhodotorulic acid	PubChem	29337	Siderophore	−2.3	2.7	*A*
Schizokinen	PubChem	3082425	Siderophore	−2.2	2.4	*A*
Enterochelin component	PubChem	151483	Siderophore component	−2.1	1.9	*B*
Vibriobactin A	PubChem	72836891	Siderophore	−2.0	2.9	*B*
Staphyloferrin A	PubChem	3035516	Siderophore	−1.9	2.0	*A*
Staphyloferrin A	PDB	SF8	Siderophore	−1.9	2.4	*B*
Deferoxamine	PubChem	2973	Siderophore	−1.8	1.5	*B*
EGTA	PubChem	6207	Small molecule	−1.7	2.5	*B*
EDDA	PubChem	61975	Small molecule	−1.7	1.7	*B*
Rhodotorulic acid	PubChem	29337	Siderophore	−1.6	3.5	*B*
δ-2-Albomycin A1	PDB	ALB	Sideromycin	−1.4	2.4	*A*
Staphyloferrin B	PubChem	46926215	Siderophore	−1.2	2.1	*A*
EDTA	PubChem	6049	Small molecule	−0.9	2.6	*A*
Coelichelin	PubChem	3247071	Siderophore	−0.5	1.2	*A*
Octaethylene glycol monomethyl ether	PDB	7PG	Small molecule	−0.5	1.5	*A*
Bis-tris propane	PDB	B3P	Small molecule	−0.2	2.5	*A*
Staphyloferrin A	PDB	SF8	Siderophore	0.0	1.0	*A*

**Table:** Rejected substrates.

Substrate	PubChem/PDB	ID	Type	Energy (Δ*G*)	Distance (Å)	Molecule
Citric acid	PubChem	311	Small molecule	REJECTED		*B*
EDTA	PubChem	6049	Small molecule	REJECTED		*B*
Deferrioxamine E	PubChem	161532	Siderophore	REJECTED		*B*
Colimycin M	PubChem	216258	Sideromycin	REJECTED		*A*
Colimycin M	PubChem	216258	Sideromycin	REJECTED		*B*
Micacocidin A	PubChem	492645	Siderophore	REJECTED		*B*
Schizokinen	PubChem	3082425	Siderophore	REJECTED		*B*
Coelichelin	PubChem	3247071	Siderophore	REJECTED		*B*
Pyoverdin	PubChem	5289234	Siderophore	REJECTED		*A*
Danoxamine	PubChem	11181103	Siderophore	REJECTED		*A*
Danoxamine	PubChem	11181103	Siderophore	REJECTED		*B*
Pyochelin isomer	PubChem	23637949	Siderophore	REJECTED		*A*
Desferrithiocin	PubChem	23694970	Siderophore	REJECTED		*A*
Desferrithiocin	PubChem	23694970	Siderophore	REJECTED		*B*
Desferoxamine B	PubChem	24883429	Siderophore	REJECTED		*A*
Desferoxamine B	PubChem	24883429	Siderophore	REJECTED		*B*
PDTC	PubChem	25201575	Siderophore	REJECTED		*A*
PDTC	PubChem	25201575	Siderophore	REJECTED		*B*
Pyochelin isomer	PubChem	46173425	Siderophore	REJECTED		*A*
Staphyloferrin B	PubChem	46926215	Siderophore	REJECTED		*A*
Vibriobactin	PubChem	50909840	Siderophore	REJECTED		*A*
Vibriobactin	PubChem	50909840	Siderophore	REJECTED		*B*
Pyochelin isomer	PubChem	54579907	Siderophore	REJECTED		*A*
EDTA	PubChem	56840845	Small molecule	REJECTED		*A*
EDTA	PubChem	56840845	Small molecule	REJECTED		*B*
Vibriobactin A	PubChem	72836891	Siderophore	REJECTED		*A*
Aminochelin	PubChem	85550078	Siderophore	REJECTED		*A*
Aminochelin	PubChem	85550078	Siderophore	REJECTED		*B*
Coprogen	PubChem	90659013	Siderophore	REJECTED		*A*
Coprogen	PubChem	90659013	Siderophore	REJECTED		*B*
Microcin B17	PubChem	101097383	Sideromycin	REJECTED		*A*
Microcin B17	PubChem	101097383	Sideromycin	REJECTED		*B*
Desferrithiocin	PubChem	101609363	Siderophore	REJECTED		*A*
Desferrithiocin	PubChem	101609363	Siderophore	REJECTED		*B*
Benzamide derivative	PubChem	101679548	Small molecule	REJECTED		*A*
Benzamide derivative	PubChem	101679548	Small molecule	REJECTED		*B*
Mycobactin S	PubChem	101705627	Siderophore	REJECTED		*A*
Mycobactin S	PubChem	101705627	Siderophore	REJECTED		*B*
Salmochelin S4	PubChem	101763507	Siderophore	REJECTED		*B*
Salmochelin S2	PubChem	101862321	Siderophore	REJECTED		*A*
Salmochelin S2	PubChem	101862321	Siderophore	REJECTED		*A*
Microcin C7	PubChem	101929386	Sideromycin	REJECTED		*A*
Microcin C7	PubChem	101929386	Sideromycin	REJECTED		*B*
Micacocidin	PubChem	101948282	Siderophore	REJECTED		*A*
Pyoverdin C	PubChem	102122857	Siderophore	REJECTED		*A*
Pyoverdin D	PubChem	102122858	Siderophore	REJECTED		*A*
Pyoverdin D	PubChem	102122858	Siderophore	REJECTED		*B*
Coprogen derivative	PubChem	102315087	Siderophore	REJECTED		*A*
Anguibactin	PubChem	121231152	Siderophore	REJECTED		*B*
Ferrioxamine E	PDB	6L0	Siderophore	REJECTED		*A*
Ferrioxamine E	PDB	6L0	Siderophore	REJECTED		*B*
Octaethylene glycol monomethyl ether	PDB	7PG	Small molecule	REJECTED		*B*
Bis-tris propane	PDB	B3P	Small molecule	REJECTED		*B*
Enterobactin component	PDB	DBH	Siderophore component	REJECTED		*A*
Enterobactin component	PDB	DBH	Siderophore component	REJECTED		*B*
RPR209685	PDB	RRR	Small molecule	REJECTED		*B*

**Table 3:** Transcription-factor binding-site predictions Transcription-factor binding site (TFBS) predictions in Hmu, Yfu, Yfe and Yiu promoters. PMW (species) indicates the transcription factor that was detected and the species from which the TFBS sequence was determined. Start and end positions indicate the positions in the promoter containing the TFBS. Strand indicates which strand contains the TFBS. Score indicates a similarity score between the promoter and the TFBS position weight matrix. Sequence indicates the nucleotides in the promoter that correspond to the TFBS.

	PWM (species)	Start position	End position	Strand	Score	Sequence
Hmu	OxyR (SELEX) | *E. coli* (strain K12)	2	47	−	13.62	ACAAAATGGATTACCGGATGAATGATTTCAGACTAACTTTTTTTCA
	CspA | *E. coli* (strain K12)	95	99	+	10	CCAAT
	GcvA | *E. coli* (strain K12)	102	106	−	10	CTAAT
Yfu	OxyR (SELEX) | *E. coli* (strain K12)	190	235	+	13.44	GAAATATTCAGATAACAATGATAATCATTTTTATTACCATAATTCG
	OxyR (SELEX) | *E. coli* (strain K12)	39	84	−	13.17	ATTATATGAAGAGTACCGGCTTTAACGGCATTTTCCTGTTTGTTCA
	CspA | *E. coli* (strain K12)	104	108	+	10	CCAAT
	GcvA | *E. coli* (strain K12)	175	179	−	10	CTAAT
Yfe	Fur (18-mer) | *E. coli* (strain K12)	170	187	−	28.77	AAAATGATTATCAATACC
	OmpR (C box)| *E. coli* (strain K12)	28	37	+	12.14	TGTAGCATAT
	CpxR | *E. coli* (strain K12)	113	128	+	12.13	AGTAACTATTGGTAAG
	CspA | *E. coli* (strain K12)	120	124	−	10	CCAAT
	GcvA | *E. coli* (strain K12)	165	169	+	10	CTAAT
Ysu	LexA | *E. coli* (strain K12)	33	48	+	10.45	TTGGCAAAAGATACAG
Yiu	OxyR (SELEX) | *E. coli* (strain K12)	27	72	−	13.61	TTGATAAGTATTATCATTTGCTTTATTGTTAGCGCCATCTTATGGG
	OxyR (SELEX) | *E. coli* (strain K12)	225	270	+	13.54	TTTATAGGCACTAAAGAAGGGCGATAGCGTTATCGCCCTTTCATCC
	OxyR (SELEX) | *E. coli* (strain K12)	152	197	−	13.4	ACGAAATGTGCTGGTATTGGCGCATTCTATCCGTGAACATCAGGCT
	CpxR | *E. coli* (strain K12)	96	111	+	12.66	CGTAACTTTTTGTAAG
	CpxR | *E. coli* (strain K12)	86	101	+	12.26	AGTAATTGGACGTAAC
	LexA | *E. coli* (strain K12)	199	214	−	10.47	CTGACGCCAATACCAG
	FhlA | *E. coli* (strain K12)	191	197	+	10.24	ATTTCGT
	CspA | *E. coli* (strain K12)	204	208	−	10	CCAAT
	CspA | *E. coli* (strain K12)	178	182	+	10	CCAAT
	CspA | *E. coli* (strain K12)	130	134	−	10	CCAAT
	GcvA | *E. coli* (strain K12)	221	225	+	10	CTAAT
	GcvA | *E. coli* (strain K12)	145	149	+	10	CTAAT
